# Comparison of clinical characteristics and predictive performance of severity scoring systems in hypertriglyceridemic versus non-hypertriglyceridemic acute pancreatitis: a retrospective cohort study

**DOI:** 10.20945/2359-4292-2026-0103

**Published:** 2026-09-12

**Authors:** Zhen Chen, Min-xing Liu, Zhen-yu Chu, Li-min Jia

**Affiliations:** 1 Department of Emergency, The First Affiliated Hospital of Soochow University, Suzhou, China; 2 Department of Emergency and Critical Care Medicine, The Affiliated Suzhou Hospital of Nanjing Medical University, Suzhou Municipal Hospital, Gusu School of Nanjing Medical University, Suzhou, China

**Keywords:** hypertriglyceridemic acute pancreatitis, clinical characteristics, BISAP, Modified CT Severity Index, severity scoring systems, retrospective cohort study

## Abstract

**Objective:**

To compare early clinical and biochemical characteristics of hypertriglyceridemic acute pancreatitis (HTG-AP) and non-hypertriglyceridemic acute pancreatitis (NHTG-AP), and to evaluate the predictive performance of commonly used severity scoring systems for adverse in-hospital outcomes.

**Subjects and methods:**

This single-center retrospective cohort study included 268 consecutive adults with acute pancreatitis admitted from January to December 2023. Patients were classified as HTG-AP (n = 128) or NHTG-AP (n = 140) according to predefined etiological criteria. Demographic data, laboratory results within 24 hours, and six scoring systems, including BISAP, MCTSI, modified Marshall score, Ranson criteria, qSOFA, and HAPS, were analyzed. The primary endpoint was a composite adverse outcome comprising persistent organ failure, pancreatic or peripancreatic necrosis requiring intervention, intensive care unit stay longer than 7 days, or in-hospital mortality. Predictors were identified using LASSO-selected multivariable logistic regression and assessed by temporal validation, receiver operating characteristic analysis, calibration, and bootstrap resampling.

**Results:**

Compared with NHTG-AP, HTG-AP patients were younger, had higher body mass index and recurrence rates, showed greater systemic inflammation and atherogenic dyslipidemia, and had lower free thyroxine levels. Composite adverse outcomes were more frequent in HTG-AP than in NHTG-AP. BISAP and MCTSI were independent predictors of adverse outcomes. The combined BISAP-MCTSI model showed good discrimination in derivation and validation cohorts and outperformed Ranson, qSOFA, and HAPS after Bonferroni correction.

**Conclusion:**

HTG-AP represents a distinct clinical phenotype with worse in-hospital outcomes. A combined BISAP-MCTSI model may improve early risk stratification and warrants prospective validation.

## INTRODUCTION

Acute pancreatitis (AP), a common and potentially life-threatening gastrointestinal disorder, involves inflammation of the pancreatic tissue. Globally, the incidence of AP has ranged from 13 to 45 cases per 100,000 population annually and has continued to rise, imposing substantial clinical and economic burdens on healthcare systems (^1^). Acute pancreatitis etiologies are diverse; the most common causes have included gallstone disease, excessive alcohol consumption, hypertriglyceridemia, and drug-induced injury (^2^). Among these, hypertriglyceridemic acute pancreatitis (HTG-AP) has garnered increasing attention as a distinct clinical subtype, particularly given its rising prevalence among younger individuals and patients with metabolic syndrome (^3^). A serum triglyceride (TG) level exceeding 11.3 mmol/L (1000 mg/dL) typically defines HTG-AP, and its incidence has paralleled the global surge in obesity, diabetes, and dyslipidemia (^4^). Unlike biliary or alcoholic pancreatitis, HTG-AP exhibits unique pathophysiological mechanisms, including elevated circulating chylomicrons, free fatty acid-induced cytotoxicity, and pancreatic microvascular ischemia (^5^). These metabolic derangements amplify systemic inflammatory responses and may predispose patients to more severe disease trajectories. Elucidating the distinct clinical and biochemical profiles of HTG-AP compared to non-hypertriglyceridemic acute pancreatitis (NHTG-AP) is therefore essential to refine diagnostic precision and optimize early therapeutic interventions.

Early and accurate AP severity assessment is paramount to guide clinical decision-making, including monitoring intensity, fluid resuscitation strategies, and escalation to critical care (^6^). Investigators have developed and validated multiple scoring systems to predict AP severity and prognosis. These include the bedside index for severity in acute pancreatitis (BISAP), the modified computed tomography severity index (MCTSI), the modified Marshall score, Ranson’s criteria, the quick sequential organ failure assessment (qSOFA), and the harmless acute pancreatitis score (HAPS) (^7^–^9^). Each tool stratifies patients by risk using distinct clinical, laboratory, or radiological parameters. However, the applicability and predictive accuracy of these scoring systems across different AP etiological subtypes, particularly HTG-AP, have remained insufficiently characterized. Previous studies developed and validated most existing scoring models predominantly in cohorts with biliary or alcoholic pancreatitis; this potentially limits their generalizability to HTG-AP, which exhibits unique metabolic and inflammatory phenotypes. Moreover, small sample sizes, heterogeneous outcome definitions, and a lack of standardized temporal assessment protocols have constrained previous comparative studies. These limitations have generated diagnostic uncertainty, potentially leading to suboptimal risk stratification or resource allocation for patients with HTG-AP (^10^). Establishing more robust risk stratification strategies for HTG-AP thus requires a systematic comparative evaluation of these scoring systems within distinct etiological subgroups using standardized methodology and composite clinical endpoints.

Despite growing recognition of HTG-AP as a distinct clinical entity, several critical knowledge gaps persist. First, although prior studies have documented differences in lipid profiles between HTG-AP and other etiologies, the comprehensive characterization of a broader spectrum of metabolic derangements, (e.g., alterations in glucose homeostasis, thyroid function, and inflammatory cascade activation) remains incomplete. Second, investigators have not rigorously evaluated the comparative predictive performance of commonly utilized severity scoring systems specifically within populations with HTG-AP while adequately controlling for temporal bias and confounding related to etiological heterogeneity. Third, existing prognostic models may succumb to circular reasoning when employing organ dysfunction scores to predict outcomes defined by organ failure itself; avoiding this requires adopting composite clinical endpoints that encompass the full range of adverse outcomes. Lastly, the notably high recurrence rate observed in HTG-AP and its relationship to the baseline metabolic phenotype warrant further investigation to inform evidence-based secondary prevention strategies.

Given the above, we sought to (^1^) to systematically compare early clinical, metabolic, and biochemical characteristics between patients with HTG-AP and those with NHTG-AP who were admitted within 24 hours of symptom onset, and (^2^) evaluate and compare the predictive accuracy of six commonly used severity scoring systems (i.e., BISAP, MCTSI, modified Marshall score, Ranson criteria, qSOFA, and HAPS) for a predefined composite adverse in-hospital outcome, determining whether a combined scoring approach offered superior risk stratification across etiological subtypes.

## SUBJECTS AND METHODS

### Study design and setting

This single-center retrospective cohort study was conducted at the First Affiliated Hospital of Soochow University (China). Consecutive adult patients admitted with AP between January 1 and December 31, 2023, were screened. The primary analysis included patients admitted within 24 hours of symptom onset; a sensitivity analysis included admissions within 72 hours. For temporal validation, patients admitted from January to October 2023 formed the derivation cohort (n = 214, ~80%), and those admitted from November to December 2023 formed the temporal validation cohort (n = 54, ~20%). The institutional review board approved the study, and informed consent was waived due to the retrospective design.

### Patient selection and eligibility criteria

Acute pancreatitis was diagnosed according to the 2012 Revised Atlanta Classification (^11^), requiring at least two of the following: (^1^) characteristic abdominal pain; (^2^) serum amylase or lipase ≥3× the upper limit of normal; (^3^) imaging findings consistent with AP. Inclusion criteria comprised age ≥18 years; admission within the predefined time window; TG measured within 24 hours of admission prior to lipid-lowering therapy; and sufficient data to calculate severity scores and ascertain outcomes. Exclusion criteria included chronic pancreatitis; a history of pancreatic surgery; malignancy-associated pancreatitis; pregnancy; transfer from another institution with delayed presentation; and incomplete records precluding score calculation or outcome assessment. For recurrent admissions, only the index admission was analyzed.

### Etiological classification

HTG-AP was defined as a TG level ≥11.3 mmol/L (1000 mg/dL) measured within 24 hours of admission in the absence of definitive alternative etiologies, or a TG level of 5.65–11.3 mmol/L (500–1000 mg/dL) with lipemic serum and the exclusion of other causes. NHTG-AP etiologies included biliary, alcohol-related, post-endoscopic retrograde cholangiopancreatography, drug-induced, and idiopathic pancreatitis, diagnosed based on standard criteria. In patients with multiple potential etiological factors, the predominant cause was determined using a hierarchical adjudication protocol. First, a biliary etiology was considered definitive if gallstones or biliary sludge were confirmed on imaging (ultrasound, computed tomography [CT], or magnetic resonance cholangiopancreatography) with concordant elevation of hepatobiliary enzymes (ALT >3× the upper limit of normal within 24 hours of admission), regardless of concomitant TG levels. Second, alcohol-related pancreatitis was diagnosed given documented significant alcohol consumption (>50 g/day for >5 years or a binge episode within 48 hours prior to symptom onset) and no identified biliary cause, even if TG levels were mildly elevated. Third, HTG-AP was diagnosed only when (a) serum TG were ≥11.3 mmol/L (1000 mg/dL), or (b) TG were 5.65–11.3 mmol/L (500–1000 mg/dL) with lipemic serum, and no definitive biliary, alcohol-related, or other identifiable cause was present. Cases with competing etiologies that could not be resolved by these criteria (e.g., TG 5.65–11.3 mmol/L with concurrent moderate alcohol use but no clear biliary disease) were independently reviewed by two senior gastroenterologists blinded to clinical outcomes. Disagreements were resolved by consensus involving a third adjudicator. Inter-rater agreement was excellent (Cohen’s κ = 0.89, 95% confidence interval [CI]: 0.82–0.96). A total of 32 cases (10.7% of the final cohort) required formal adjudication; of these, 18 were classified as HTG-AP and 14 as NHTG-AP.

### Data collection

We extracted demographics, comorbidities, clinical presentation, and vital signs from electronic medical records using a standardized form. We recorded laboratory values obtained within the first 24 hours, including inflammatory markers, metabolic indices, lipid profiles, renal and liver function, and thyroid function tests. Patients underwent contrast-enhanced CT according to routine clinical practice.

### Severity scoring systems

This study evaluated six commonly used severity scoring systems. Supplementary Table S1 provides detailed definitions, individual components, score ranges, interpretation thresholds, and assessment time windows, which we summarize below.

1) Bedside index for severity in acute pancreatitis (BISAP): A 5-point clinical score assessed within the first 24 hours of admission, comprising five dichotomous variables: blood urea nitrogen (BUN) >25 mg/dL (>7.14 mmol/L), impaired mental status (Glasgow coma scale [GCS] <15), the presence of systemic inflammatory response syndrome (SIRS; defined as ≥2 of the following: temperature >38 or <36 °C, heart rate >90 beats/min, respiratory rate >20 breaths/min or PaCO_2_ <32 mmHg, white blood cell count >12,000 or <4,000/mm^3^), age >60 years, and pleural effusion detected on imaging. A score ≥3 indicates a high risk for severe disease and in-hospital mortality (^12^).
2) Modified computed tomography severity index (MCTSI): A 10-point imaging-based score derived from contrast-enhanced computed tomography findings, assessed when the imaging procedure was performed according to routine clinical practice (typically within 72 hours of admission). The MCTSI comprises three domains: degree of pancreatic inflammation (0, 2, or 4 points), extent of pancreatic necrosis (0 = none, 2 = ≤30%, 4 = >30%), and the presence of extrapancreatic complications (2 points for any of the following: pleural effusion, ascites, vascular complications, or extrapancreatic parenchymal complications). A total score ≥4 indicates moderate-to-severe disease (^13^).
3) Modified Marshall scoring system: A physiological score evaluating organ dysfunction across three systems: respiratory (PaO_2_/FiO_2_ ratio), renal (serum creatinine), and cardiovascular (systolic blood pressure, with or without response to fluid resuscitation). The system scores each category from 0 to 4. A score ≥2 in any single system indicates organ failure. We initially calculated scores using the worst values recorded within the first 24 hours of admission and reassessed them at 48 hours to determine persistence. In accordance with the Revised Atlanta Classification, we defined persistent organ failure as organ failure lasting ≥48 hours (^14^).
4) Ranson’s criteria: An 11-point score incorporating two distinct assessment time points. The score evaluates five criteria at admission: age >55 years, white blood cell count >16,000/mm^3^, blood glucose >200 mg/dL (>11.1 mmol/L), lactate dehydrogenase >350 IU/L, and aspartate aminotransferase >250 IU/L. Six additional criteria apply at 48 hours: a hematocrit decrease >10 percentage points, a BUN increase >5 mg/dL (>1.79 mmol/L), serum calcium <8 mg/dL (<2.0 mmol/L), PaO_2_ <60 mmHg, base deficit >4 mEq/L, and estimated fluid sequestration >6 L. A total score ≥3 predicts severe disease. We collected the 48-hour components when available, according to the original scoring definition (^15^).
5) Quick sequential organ failure assessment (qSOFA): A 3-point bedside screening score assessed within the first 24 hours of admission, comprising three dichotomous criteria: altered mental status (GCS <15), systolic blood pressure ≤100 mmHg, and respiratory rate ≥22 breaths/min. A score ≥2 suggests an elevated risk for a poor clinical outcome (^16^).
6) Harmless acute pancreatitis score (HAPS): A rule-out score assessed within the first 24 hours of admission, designed to identify patients likely to have a non-severe clinical course. The score comprises three criteria: the absence of rebound abdominal tenderness or guarding, a normal hematocrit (30–44%), and normal serum creatinine (≤2.0 mg/dL or ≤177 µmol/L). A score of 3 (all three criteria fulfilled) predicts a harmless course with a high negative predictive value (^17^).

For all scoring systems, we calculated scores using the worst clinical and laboratory values obtained within the specified assessment time windows. When multiple measurements were available within the assessment window, we selected the most abnormal value to ensure we captured peak disease severity.

### Outcomes

The primary outcome was an in-hospital composite adverse endpoint, defined as any of the following: (^1^) persistent organ failure (modified Marshall score ≥2 for ≥48 hours); (^2^) pancreatic or peripancreatic necrosis requiring continuous intervention (percutaneous, endoscopic, or surgical); (^3^) intensive care unit (ICU) stay >7 days; or (^4^) in-hospital mortality. Secondary outcomes included individual components of the composite endpoint, pancreatic necrosis, ICU admission, and length of stay.

The composite adverse outcome was selected a priori based on the following rationale. First, individual severe outcomes in AP (mortality, persistent organ failure, necrotizing pancreatitis requiring intervention) are relatively infrequent in moderate-sized cohorts, which limits statistical power for single-endpoint analyses. Second, a composite endpoint encompassing the full spectrum of clinically meaningful adverse events captures the aggregate burden of severe disease and aligns with endpoints used in recent severity studies. Third, to prevent circular reasoning, the composite outcome was deliberately constructed so it does not simply recapitulate a single scoring system’s definition (e.g., modified Marshall ≥2 alone); rather, it integrates multiple independent clinical domains. To facilitate clinical interpretation regarding their relative contributions, the individual components of the composite outcome are also reported separately as secondary endpoints (Table 1).

**Table 1.** Baseline demographic, clinical, and comorbidity characteristics and clinical outcomes of patients with acute pancreatitis stratified by etiology

Variable	Total (n=268)	HTG-AP (n=128)	NHTG-AP (n=140)	Test statistic	*p*-Value	Effect size
Demographics						
Age (years), mean ± SD	43.25 ± 12.38	39.84 ± 10.52	46.38 ± 13.15	t = −4.321	<0.001*	0.53^a^
Male sex, n (%)	189 (70.5)	102 (79.7)	87 (62.1)	χ^2^ = 10.127	0.001*	0.19^b^
BMI (kg/m^2^), mean ± SD	26.47 ± 4.32	28.15 ± 4.68	24.96 ± 3.51	t = 6.189	<0.001*	0.76^a^
Clinical presentation						
Time from onset to admission (hours), median (IQR)	18.5 (12.0–30.0)	16.0 (10.0–26.0)	21.0 (14.0–36.0)	U = 7342	0.009*	0.21^c^
Abdominal pain on admission, n (%)	265 (98.9)	127 (99.2)	138 (98.6)	Fisher's exact	1.000	0.03^b^
Nausea/vomiting, n (%)	218 (81.3)	108 (84.4)	110 (78.6)	χ^2^ = 1.523	0.217	0.08^b^
Fever (>38 °C), n (%)	87 (32.5)	48 (37.5)	39 (27.9)	χ^2^ = 2.936	0.087	0.10^b^
Primary Etiology (mutually exclusive)†				χ^2^ = 162.34	<0.001*	0.78^b^
Hypertriglyceridemia, n (%)	128 (47.8)	128 (100.0)	0 (0.0)	—	—	—
Biliary, n (%)	74 (27.6)	0 (0.0)	74 (52.9)	—	—	—
Alcohol-related, n (%)	35 (13.1)	0 (0.0)	35 (25.0)	—	—	—
Post-ERCP/biliary surgery, n (%)	16 (6.0)	0 (0.0)	16 (11.4)	—	—	—
Drug-induced, n (%)	7 (2.6)	0 (0.0)	7 (5.0)	—	—	—
Idiopathic, n (%)	8 (3.0)	0 (0.0)	8 (5.7)	—	—	—
Precipitating/Contributing Factors (non-exclusive)^‡^						
Overeating/binge eating, n (%)	104 (38.8)	67 (52.3)	37 (26.4)	χ^2^ = 18.72	<0.001*	0.26^b^
Concurrent alcohol use, n (%)	58 (21.6)	18 (14.1)	40 (28.6)	χ^2^ = 8.45	0.004*	0.18^b^
Concurrent biliary disease, n (%)	14 (5.2)	8 (6.3)	6 (4.3)	χ^2^ = 0.529	0.467	0.04^b^
Any coexisting etiological factor, n (%)	47 (17.5)	28 (21.9)	19 (13.6)	χ^2^ = 3.195	0.074	0.11^b^
Comorbidities						
Recurrent pancreatitis, n (%)	78 (29.1)	51 (39.8)	27 (19.3)	χ^2^ = 13.945	<0.001*	0.23^b^
Diabetes mellitus, n (%)	92 (34.3)	58 (45.3)	34 (24.3)	χ^2^ = 13.483	<0.001*	0.22^b^
Hypertension, n (%)	98 (36.6)	52 (40.6)	46 (32.9)	χ^2^ = 1.823	0.177	0.08^b^
Known dyslipidemia, n (%)	145 (54.1)	96 (75.0)	49 (35.0)	χ^2^ = 42.687	<0.001*	0.40^b^
Coronary artery disease, n (%)	36 (13.4)	21 (16.4)	15 (10.7)	χ^2^ = 1.928	0.165	0.09^b^
Chronic kidney disease, n (%)	18 (6.7)	10 (7.8)	8 (5.7)	χ^2^ = 0.502	0.478	0.04^b^
Lifestyle Factors						
Current/former smoker, n (%)	127 (47.4)	67 (52.3)	60 (42.9)	χ^2^ = 2.546	0.111	0.10^b^
Regular alcohol consumption, n (%)	129 (48.1)	64 (50.0)	65 (46.4)	χ^2^ = 0.360	0.548	0.04^b^
Initial severity assessment						
SIRS criteria met on admission, n (%)	156 (58.2)	84 (65.6)	72 (51.4)	χ^2^ = 5.734	0.017*	0.15^b^
Organ failure on admission, n (%)	42 (15.7)	26 (20.3)	16 (11.4)	χ^2^ = 4.228	0.040*	0.13^b^
Respiratory failure, n (%)	28 (10.4)	18 (14.1)	10 (7.1)	χ^2^ = 3.542	0.060	0.12^b^
Cardiovascular failure, n (%)	19 (7.1)	12 (9.4)	7 (5.0)	χ^2^ = 2.013	0.156	0.09^b^
Renal failure, n (%)	15 (5.6)	10 (7.8)	5 (3.6)	χ^2^ = 2.465	0.116	0.10^b^
ICU admission required, n (%)	68 (25.4)	39 (30.5)	29 (20.7)	χ^2^ = 3.563	0.059	0.12^b^
Disease Severity (Revised Atlanta)^§^				χ^2^ = 12.845	0.002*	0.22^b^
Mild AP, n (%)	168 (62.7)	72 (56.3)	96 (68.6)	—	—	—
Moderately severe AP, n (%)	58 (21.6)	28 (21.9)	30 (21.4)	—	—	—
Severe AP, n (%)	42 (15.7)	28 (21.9)	14 (10.0)	—	—	—
Clinical Outcomes						
Composite adverse outcome, n (%)^‖^	57 (21.3)	36 (28.1)	21 (15.0)	χ^2^ = 6.980	0.008*	0.16^b^
Components (non-exclusive):						
Persistent organ failure (≥48 h), n (%)	36 (13.4)	24 (18.8)	12 (8.6)	χ^2^ = 5.867	0.015*	0.15^b^
Necrosis requiring intervention, n (%)	31 (11.6)	19 (14.8)	12 (8.6)	χ^2^ = 2.663	0.103	0.10^b^
ICU stay >7 days, n (%)	28 (10.4)	19 (14.8)	9 (6.4)	χ^2^ = 5.172	0.023*	0.14^b^
In-hospital mortality, n (%)	6 (2.2)	4 (3.1)	2 (1.4)	Fisher's exact	0.426	0.06^b^
Other outcomes:						
Pancreatic necrosis (any), n (%)	38 (14.2)	24 (18.8)	14 (10.0)	χ^2^ = 4.389	0.036*	0.13^b^
Infected necrosis, n (%)	15 (5.6)	10 (7.8)	5 (3.6)	χ^2^ = 2.465	0.116	0.10^b^
Any intervention required, n (%)	31 (11.6)	19 (14.8)	12 (8.6)	χ^2^ = 2.663	0.103	0.10^b^
Length of hospital stay (days), median (IQR)	9.0 (6.0–14.0)	10.0 (7.0–16.0)	8.0 (5.0–12.0)	U = 7256	0.007*	0.22^c^

HTG-AP: hypertriglyceridemic acute pancreatitis; NHTG-AP: non-hypertriglyceridemic acute pancreatitis; BMI: body mass index; IQR: interquartile range; ERCP: endoscopic retrograde cholangiopancreatography; SIRS: systemic inflammatory response syndrome; ICU: intensive care unit; AP: acute pancreatitis.

* Statistically significant at *p* < 0.05 (two-sided).

a Effect size measures: Cohen’s d for independent-samples t-tests;

b Cramér’s V (φc) for χ² and Fisher’s exact tests;

c rank-biserial correlation (r) for Mann-Whitney U tests.

† Primary etiology was assigned as a mutually exclusive category using the hierarchical adjudication protocol described in the Methods section. By definition, all patients in the HTG-AP group had hypertriglyceridemia as the primary etiology, and all patients in the NHTG-AP group had a non-hypertriglyceridemic primary etiology. The overall χ^2^ test compares the distribution across all primary etiology categories.

‡ Precipitating/contributing factors are non-mutually exclusive and reflect coexisting etiological risk factors not classified as the primary etiology. A single patient may have more than one contributing factor. The detailed distribution of overlapping etiologies is provided in Supplementary Table S3.

§ Disease severity was classified according to the Revised Atlanta Classification (2012): mild (no organ failure, no local/systemic complications), moderately severe (transient organ failure <48 h and/or local complications), severe (persistent organ failure ≥48 h).

II Composite adverse outcome was defined as any of the following during the index hospitalization: (^1^) persistent organ failure (Modified Marshall score ≥2 in any organ system for ≥48 hours); (^2^) pancreatic or peripancreatic necrosis requiring intervention (percutaneous drainage, endoscopic necrosectomy, or surgical necrosectomy); (^3^) ICU stay >7 days; or (^4^) in-hospital mortality. Components are non-mutually exclusive; among 57 patients meeting the composite endpoint, 32 (56.1%) satisfied two or more criteria.

### Statistical analysis

We summarized continuous variables as means ± standard deviation (SD) or medians (interquartile ranges [IQRs]), depending on their distributions as assessed by the Shapiro-Wilk test, and compared them between groups using independent-samples t tests or Mann-Whitney U tests, as appropriate. We expressed categorical variables as frequencies (percentages) and compared them using χ^2^ tests or Fisher’s exact tests. For multiple laboratory comparisons (Table 2), we applied the Benjamini-Hochberg false discovery rate procedure to control for multiplicity.

**Table 2.** Comparative laboratory and biochemical parameters on admission in patients with hypertriglyceridemic versus non-hypertriglyceridemic acute pancreatitis

Variable	Total (n=268)	HTG-AP (n=128)	NHTG-AP (n=140)	Mean difference (95% CI)	Test statistic	*p*-Value	Effect size
Pancreatic enzymes							
Serum amylase (U/L), median (IQR)	856 (412-1654)	792 (385-1521)	918 (456-1742)	-	U = 8234	0.156	0.11
Serum lipase (U/L), median (IQR)	1245 (587-2876)	1156 (542-2654)	1328 (618-3021)	-	U = 8456	0.234	0.09
Inflammatory Markers							
WBC count (×10⁹/L), mean ± SD	12.85 ± 4.67	13.94 ± 5.12	11.86 ± 4.02	2.08 (0.89-3.27)	t = 3.456	0.001*	0.45
Neutrophil percentage (%), mean ± SD	78.5 ± 9.8	81.2 ± 8.9	76.1 ± 10.2	5.1 (2.4-7.8)	t = 3.287	0.001*	0.53
CRP (mg/L), median (IQR)	54.3 (18.6-126.5)	86.7 (32.4-168.2)	38.9 (12.8-94.3)	-	U = 6234	<0.001*	0.34
PCT (ng/mL), median (IQR)	0.42 (0.12-1.86)	0.58 (0.18-2.34)	0.31 (0.09-1.45)	-	U = 7123	0.018*	0.19
Lipid profile							
Triglycerides (mmol/L), median (IQR)	5.82 (2.14-18.45)	22.67 (14.32-36.84)	2.08 (1.32-4.56)	-	U = 1245	<0.001*	0.92
Total cholesterol (mmol/L), mean ± SD	6.24 ± 2.87	8.15 ± 3.24	4.52 ± 1.68	3.63 (2.95-4.31)	t = 10.234	<0.001*	1.41
HDL-C (mmol/L), mean ± SD	0.96 ± 0.34	0.78 ± 0.28	1.12 ± 0.32	-0.34 (-0.42-0.26)	t = -8.456	<0.001*	1.12
LDL-C (mmol/L), mean ± SD	3.45 ± 1.56	4.28 ± 1.82	2.71 ± 0.98	1.57 (1.19-1.95)	t = 8.123	<0.001*	1.04
TG/HDL-C ratio, median (IQR)	6.18 (2.34-19.87)	28.45 (18.34-46.72)	1.86 (1.18-4.05)	-	U = 876	<0.001*	0.95
LDL/HDL-C ratio, mean ± SD	3.72 ± 1.85	5.68 ± 2.14	2.43 ± 0.96	3.25 (2.81-3.69)	t = 11.567	<0.001*	1.96
Liver function tests							
Total bilirubin (µmol/L), median (IQR)	18.6 (10.2-34.8)	14.3 (8.7-24.5)	28.4 (15.6-52.3)	-	U = 5678	<0.001*	0.41
Direct bilirubin (µmol/L), median (IQR)	7.8 (3.2-18.6)	5.2 (2.4-12.4)	12.6 (5.8-28.7)	-	U = 5845	<0.001*	0.38
ALT (U/L), median (IQR)	98 (42-256)	68 (32-164)	142 (58-384)	-	U = 6234	<0.001*	0.35
AST (U/L), median (IQR)	86 (38-218)	58 (28-142)	126 (52-312)	-	U = 5987	<0.001*	0.37
ALP (U/L), median (IQR)	112 (78-168)	98 (72-148)	128 (86-192)	-	U = 7234	0.002*	0.25
GGT (U/L), median (IQR)	124 (56-248)	98 (48-196)	156 (72-298)	-	U = 6987	0.005*	0.22
Albumin (g/L), mean ± SD	36.8 ± 5.6	35.4 ± 5.8	38.1 ± 5.2	-2.7 (-4.0–-1.4)	t = -3.987	<0.001*	0.49
Renal function							
Serum creatinine (µmol/L), mean ± SD	78.5 ± 32.4	84.6 ± 38.7	72.9 ± 24.1	11.7 (3.2-20.2)	t = 2.734	0.007*	0.35
BUN (mmol/L), mean ± SD	6.84 ± 3.56	7.52 ± 4.12	6.23 ± 2.84	1.29 (0.34-2.24)	t = 2.678	0.008*	0.36
eGFR (mL/min/1.73 m^2^), mean ± SD	94.6 ± 24.8	89.2 ± 27.6	99.4 ± 20.8	-10.2 (-16.1–-4.3)	t = -3.456	0.001*	0.41
Metabolic parameters							
Fasting blood glucose (mmol/L), mean ± SD	9.45 ± 4.28	11.34 ± 5.12	7.76 ± 2.68	3.58 (2.54-4.62)	t = 6.789	<0.001*	0.85
HbA1c (%)	6.8 ± 1.6	7.6 ± 1.8	6.1 ± 1.2	1.5 (1.1-1.9)	t = 7.234	<0.001*	0.98
Serum calcium (mmol/L), mean ± SD	2.08 ± 0.28	2.01 ± 0.31	2.14 ± 0.24	-0.13 (-0.20–-0.06)	t = -3.678	<0.001*	0.46
Corrected calcium (mmol/L)^††^	2.18 ± 0.26	2.12 ± 0.29	2.23 ± 0.22	-0.11 (-0.18–-0.04)	t = -3.234	0.001*	0.42
Coagulation profile							
D-dimer (mg/L), median (IQR)	1.24 (0.48-3.86)	1.56 (0.62-4.52)	0.98 (0.38-3.12)	-	U = 7456	0.042*	0.16
Fibrinogen (g/L), mean ± SD	4.52 ± 1.68	4.98 ± 1.84	4.12 ± 1.45	0.86 (0.38-1.34)	t = 3.456	0.001*	0.51
PT (seconds), mean ± SD	12.8 ± 1.6	13.2 ± 1.8	12.5 ± 1.4	0.7 (0.2-1.2)	t = 2.678	0.008*	0.43
APTT (seconds), mean ± SD	32.4 ± 5.8	33.6 ± 6.4	31.3 ± 5.1	2.3 (0.5-4.1)	t = 2.456	0.015*	0.39
Thyroid Function							
TSH (mIU/L), median (IQR)	1.86 (0.92-3.42)	1.68 (0.78-3.12)	2.04 (1.08-3.68)	-	U = 8123	0.124	0.12
Free T4 (pmol/L), mean ± SD	14.8 ± 3.6	12.6 ± 3.2	16.8 ± 3.4	-4.2 (-5.2–-3.2)	t = -7.892	<0.001*	1.26
Free T3 (pmol/L), mean ± SD	3.8 ± 0.9	3.2 ± 0.8	4.3 ± 0.8	-1.1 (-1.3–-0.9)	t = -8.456	<0.001*	1.38
Electrolytes							
Serum sodium (mmol/L), mean ± SD	138.6 ± 4.2	137.8 ± 4.6	139.3 ± 3.7	-1.5 (-2.6–-0.4)	t = -2.678	0.008*	0.36
Serum potassium (mmol/L), mean ± SD	3.92 ± 0.58	3.84 ± 0.62	3.99 ± 0.54	-0.15 (-0.31–0.01)	t = -1.867	0.063	0.26
Arterial blood gas^§§^							
pH, mean ± SD	7.38 ± 0.08	7.36 ± 0.09	7.40 ± 0.07	-0.04 (-0.06–-0.02)	t = -3.456	0.001*	0.49
PaO2/FiO2 ratio, mean ± SD	348 ± 86	326 ± 94	368 ± 75	-42 (-65–-19)	t = -3.678	<0.001*	0.49
Lactate (mmol/L), median (IQR)	1.8 (1.2-3.4)	2.2 (1.4-4.1)	1.5 (1.0-2.8)	-	U = 6789	0.003*	0.24

HTG-AP: hypertriglyceridemic acute pancreatitis; NHTG-AP: non-hypertriglyceridemic acute pancreatitis; IQR: interquartile range; WBC: white blood cell; CRP: C-reactive protein; PCT: procalcitonin; HDL-C: high-density lipoprotein cholesterol; LDL-C: low-density lipoprotein cholesterol; TG: triglycerides; ALT: alanine aminotransferase; AST: aspartate aminotransferase; ALP: alkaline phosphatase; GGT: gamma-glutamyl transferase; BUN: blood urea nitrogen; eGFR: estimated glomerular filtration rate; HbA1c: glycated hemoglobin; PT: prothrombin time; APTT: activated partial thromboplastin time; TSH: thyroid-stimulating hormone; T3: triiodothyronine; T4: thyroxine; PaO_2_: partial pressure of oxygen; FiO_2_: fraction of inspired oxygen.

To identify independent predictors of the composite adverse outcome from among the six candidate severity scores (BISAP, MCTSI, modified Marshall, Ranson, qSOFA, and HAPS), we employed a two-stage modeling strategy. In the first stage, we performed least absolute shrinkage and selection operator (LASSO) logistic regression to simultaneously address multicollinearity and variable selection. We standardized all continuous severity scores to zero mean and unit variance prior to penalization to ensure equal weighting during regularization. We selected the regularization parameter λ using 10-fold cross-validation, choosing the λ within one standard error of the minimum deviance (λ_1se_) to favor a parsimonious model and reduce overfitting risk. At the optimal λ_1se_ = 0.042, the model retained BISAP and MCTSI with non-zero coefficients (LASSO-penalized β: BISAP = 1.86, MCTSI = 0.43) and shrank the remaining four scores to zero. In the second stage, we entered the LASSO-selected variables into an unpenalized multivariable logistic regression model for final coefficient estimation and statistical inference, as LASSO-penalized coefficients are inherently biased and do not yield valid standard errors for hypothesis testing.

We evaluated collinearity among predictors at two levels. For the final multivariable model, we calculated variance inflation factors and the condition number of the design matrix. Additionally, we constructed a pairwise Spearman correlation matrix of all six candidate severity scores to characterize the interrelationships among the scoring systems and provide context for the LASSO selection results (Supplementary Table S2).

We assessed model discrimination by calculating the area under the receiver operating characteristic curve (AUC) using DeLong’s method. We evaluated calibration using calibration plots (observed vs. predicted probabilities across deciles of predicted risk), the Hosmer-Lemeshow goodness-of-fit test, and calibration slope and intercept metrics. We performed internal validation using bootstrap resampling (2000 iterations) to estimate the optimism-corrected C-statistic. We conducted temporal validation in a held-out cohort comprising patients admitted from November to December 2023 (n = 54, ~20% of the total sample).

We performed 15 pairwise AUC comparisons among the seven models (six individual scores plus the combined BISAP-MCTSI model) using DeLong’s test. To balance the risk of type I error inflation against the potential for excessive conservatism, we applied both the Bonferroni correction (adjusted α = 0.05/15 = 0.0033) and the Benjamini-Hochberg false discovery rate procedure as a complementary sensitivity approach. We present the results under both correction methods in Table 3, allowing readers to interpret the findings under their preferred framework for multiple comparison adjustment.

**Table 3.** Comparison of clinical severity scoring systems between hypertriglyceridemic and non-hypertriglyceridemic acute pancreatitis cohorts

Scoring system	Total (n=268)	HTG-AP (n=128)	NHTG-AP (n=140)	Mean difference (95% CI)	Test statistic	*p*-Value	Effect size	AUC for composite adverse outcome (95% CI)
BISAP Score								
Total score, mean ± SD	1.24 ± 0.96	1.52 ± 1.05	0.99 ± 0.81	0.53 (0.28-0.78)	t=4.287	<0.001*	0.56	0.842 (0.768-0.916)
Score ≥3, n (%)	34 (12.7)	24 (18.8)	10 (7.1)	-	χ^2^=8.234	0.004*	0.18	-
Individual BISAP components, n (%)								
BUN >7.14 mmol/L	78 (29.1)	46 (35.9)	32 (22.9)	-	χ^2^=5.432	0.020*	0.14	-
Impaired mental status	18 (6.7)	12 (9.4)	6 (4.3)	-	Fisher’s exact	0.126	0.11	-
SIRS present	156 (58.2)	84 (65.6)	72 (51.4)	-	χ^2^=5.734	0.017*	0.15	-
Age >60 years	42 (15.7)	12 (9.4)	30 (21.4)	-	χ^2^=7.654	0.006*	0.17	-
Pleural effusion	68 (25.4)	38 (29.7)	30 (21.4)	-	χ^2^=2.456	0.117	0.10	-
Modified CT severity index (MCTSI)								
Total score, mean ± SD	2.86 ± 1.48	3.24 ± 1.62	2.52 ± 1.26	0.72 (0.34-1.10)	t=3.789	<0.001*	0.49	0.768 (0.682-0.854)
Score ≥4, n (%)	98 (36.6)	58 (45.3)	40 (28.6)	-	χ^2^=8.234	0.004*	0.18	-
MCTSI components								
Pancreatic inflammation score, median (IQR)	2 (2-4)	2 (2-4)	2 (2-4)	-	U=8234	0.234	0.09	-
Pancreatic necrosis, n (%)	38 (14.2)	24 (18.8)	14 (10.0)	-	χ^2^=4.389	0.036*	0.13	-
Extrapancreatic complications, n (%)	82 (30.6)	48 (37.5)	34 (24.3)	-	χ^2^=5.432	0.020*	0.14	-
Modified Marshall Score								
Total score, mean ± SD	1.34 ± 1.86	1.84 ± 2.24	0.89 ± 1.32	0.95 (0.48-1.42)	t=3.987	<0.001*	0.50	0.831 (0.755-0.907)
Score ≥2 (organ failure), n (%)	42 (15.7)	26 (20.3)	16 (11.4)	-	χ^2^=4.228	0.040*	0.13	-
Modified Marshall Components, mean ± SD								
Respiratory score	0.54 ± 0.92	0.72 ± 1.08	0.38 ± 0.74	0.34 (0.10-0.58)	t=2.789	0.006*	0.36	-
Cardiovascular score	0.42 ± 0.78	0.58 ± 0.94	0.28 ± 0.58	0.30 (0.09-0.51)	t=2.678	0.008*	0.37	-
Renal score	0.38 ± 0.82	0.54 ± 0.96	0.23 ± 0.64	0.31 (0.10-0.52)	t=2.789	0.006*	0.37	-
Ranson Score								
Total score, mean ± SD	2.48 ± 1.68	2.64 ± 1.78	2.34 ± 1.58	0.30 (-0.14–0.74)	t=1.345	0.180	0.18	0.698 (0.604-0.792)
Score ≥3, n (%)	98 (36.6)	52 (40.6)	46 (32.9)	-	χ^2^=1.823	0.177	0.08	-
qSOFA Score								
Total score, median (IQR)	1 (0-1)	1 (0-2)	0 (0-1)	-	U=6789	0.002*	0.25	0.724 (0.632-0.816)
Score ≥2, n (%)	58 (21.6)	38 (29.7)	20 (14.3)	-	χ^2^=9.234	0.002*	0.19	-
qSOFA Components, n (%)								
Altered mental status (GCS <15)	18 (6.7)	12 (9.4)	6 (4.3)	-	Fisher’s exact	0.126	0.11	-
Systolic BP ≤100 mmHg	68 (25.4)	42 (32.8)	26 (18.6)	-	χ^2^=7.234	0.007*	0.17	-
Respiratory rate ≥22/min	124 (46.3)	72 (56.3)	52 (37.1)	-	χ^2^=10.123	0.001*	0.19	-
HAPS Score								
Total score, mean ± SD	2.18 ± 0.56	2.08 ± 0.58	2.27 ± 0.54	-0.19 (-0.33–-0.05)	t=-2.678	0.008*	0.34	0.356‡‡ (0.264-0.448)
Score=3 (harmless), n (%)	96 (35.8)	38 (29.7)	58 (41.4)	-	χ^2^=4.123	0.042*	0.12	-
HAPS Components (absence indicates harmless), n (%)								
No rebound tenderness/guarding	192 (71.6)	86 (67.2)	106 (75.7)	-	χ^2^=2.543	0.111	0.10	-
Normal hematocrit	168 (62.7)	72 (56.3)	96 (68.6)	-	χ^2^=4.432	0.035*	0.13	-
Normal serum creatinine	224 (83.6)	102 (79.7)	122 (87.1)	-	χ^2^=2.876	0.090	0.10	-
Composite risk stratification								
Low risk (BISAP 0–1 and MCTSI 0–2)	120 (44.8%)	46 (35.9%)	74 (52.9%)	-	χ^2^=7.823	0.005*	0.17	-
Intermediate risk (BISAP ≥2 OR MCTSI 3–4, not both high)	96 (35.8%)	46 (35.9%)	50 (35.7%)	-	-	-	-	-
High risk (BISAP ≥2 AND MCTSI ≥4)	52 (19.4%)	36 (28.1%)	16 (11.4%)	-	χ^2^=12.456	<0.001*	0.22	-

HTG-AP: hypertriglyceridemic acute pancreatitis; NHTG-AP: non-hypertriglyceridemic acute pancreatitis; BISAP: Bedside Index for Severity in Acute Pancreatitis; MCTSI: Modified CT Severity Index; qSOFA: quick Sequential Organ Failure Assessment; HAPS: Harmless Acute Pancreatitis Score; BUN: blood urea nitrogen; SIRS: systemic inflammatory response syndrome; GCS: Glasgow Coma Scale; BP: blood pressure; IQR: interquartile range; AUC: area under the receiver operating characteristic curve; SAP: severe acute pancreatitis; CI: confidence interval.

* Statistically significant at *P* < 0.05 (two-sided).

As an additional sensitivity analysis, we performed propensity score matching to balance baseline characteristics between the HTG-AP and NHTG-AP groups. We applied a 1:1 nearest-neighbor matching algorithm without replacement, using a caliper width of 0.2 standard deviations of the logit of the propensity score. The covariates included in the propensity score model were age, sex, body mass index (BMI), diabetes mellitus, hypertension, and time from symptom onset to admission. We confirmed the adequacy of balance using standardized mean differences, with a target threshold of <0.10 for all matched covariates.

We conducted all statistical analyses using the software SPSS v. 26.0 (IBM Corp., USA) and R v. 4.3.1 (R Foundation for Statistical Computing, Austria). We used the R packages glmnet (LASSO regression), pROC (ROC analysis), and MatchIt (propensity score matching) for specialized analyses. All tests were two-sided, and *p* < 0.05 was considered statistically significant unless otherwise specified for multiple comparison adjustments.

## RESULTS

### Study population

**Figure 1:**
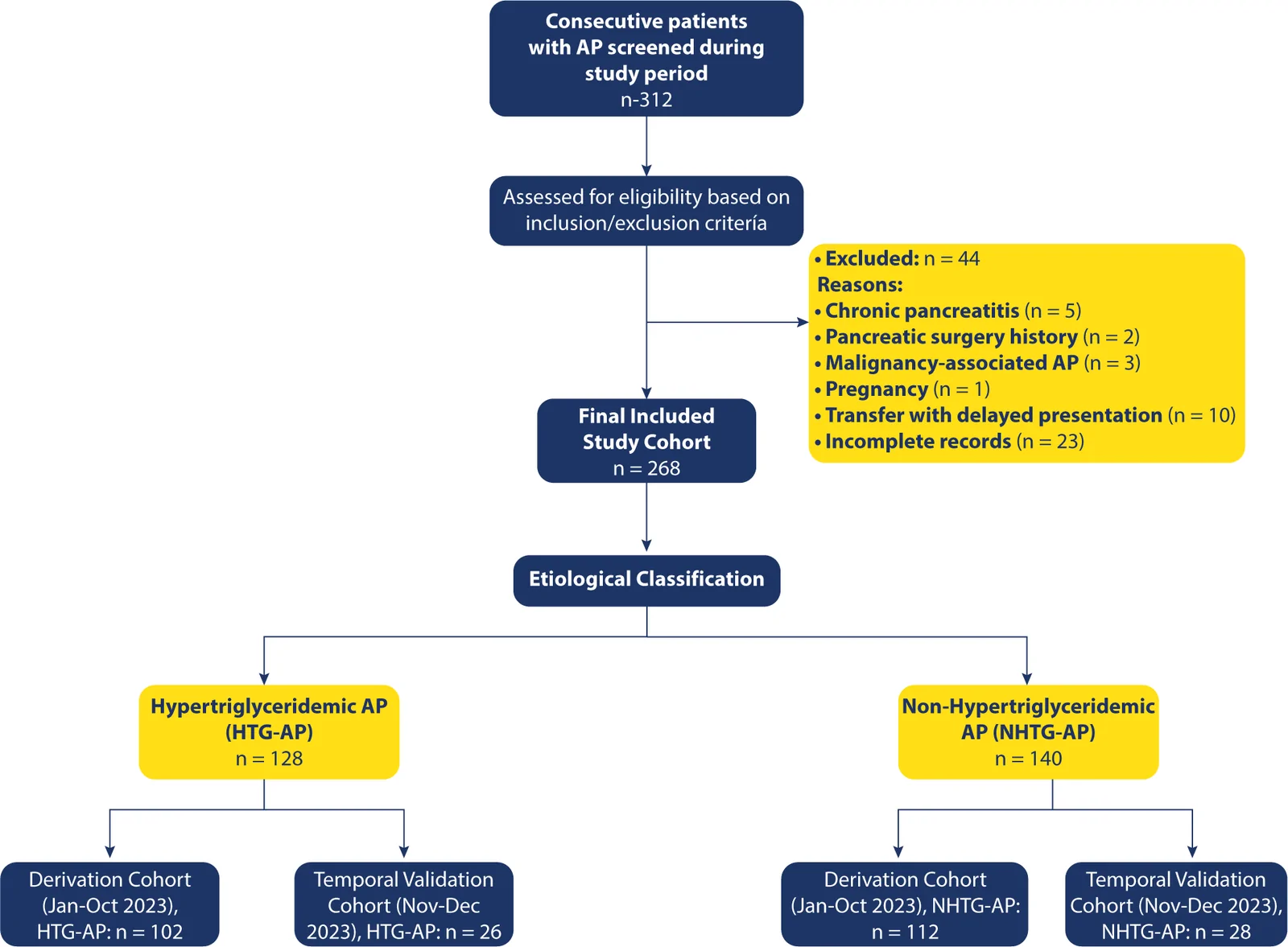
Study flow diagram and patient selection.

During the study period, we screened 312 consecutive patients with AP. Of these, 268 met the eligibility criteria and comprised the final cohort (HTG-AP, n = 128; NHTG-AP, n = 140). Figure 1 summarizes the exclusion details. Cases with competing etiologies were adjudicated with high inter-rater agreement (κ = 0.89). Table 1 presents the baseline characteristics and clinical features.

### Baseline characteristics

Patients with HTG-AP were younger (39.84 ± 10.52 vs. 46.38 ± 13.15 years; *p* < 0.001), more frequently male (79.7% vs. 62.1%; *p* = 0.001), and had a higher BMI (28.15 ± 4.68 vs. 24.96 ± 3.51 kg/m^2^; *p* < 0.001) than those with NHTG-AP. Metabolic comorbidities were more prevalent in the HTG-AP group, including diabetes mellitus (45.3% vs. 24.3%; *p* < 0.001) and known dyslipidemia (75.0% vs. 35.0%; *p* < 0.001). Recurrent pancreatitis was also more common in this group (39.8% vs. 19.3%; *p* < 0.001). On admission, patients with HTG-AP more frequently met the SIRS criteria (65.6% vs. 51.4%; *p* = 0.017) and exhibited organ failure (20.3% vs. 11.4%; *p* = 0.040) (Table 1).

We observed etiological overlap in a subset of patients. Across the entire cohort, 47 patients (17.5%) had more than one identifiable etiological risk factor. In the HTG-AP group, 28 patients (21.9%) had at least one coexisting etiological factor (18 with concomitant alcohol use, 8 with biliary disease, and 2 with both), compared with 19 patients (13.6%) in the NHTG-AP group (*p* = 0.074). A hierarchical adjudication protocol determined the predominant etiology in these cases (see the Methods section). Supplementary Table S3 details the distribution of coexisting etiological risk factors.

### Laboratory findings

Table 2 presents the comparisons of laboratory parameters obtained on admission. Patients with HTG-AP exhibited a greater inflammatory burden, indicated by a higher WBC count (13.94 ± 5.12 vs. 11.86 ± 4.02×10^9^/L; *p* = 0.001) and higher CRP levels (median 86.7 vs. 38.9 mg/L; *p* < 0.001). As expected, TG were markedly elevated in the HTG-AP group (median 22.67 vs. 2.08 mmol/L; *p* < 0.001). These patients also showed lower HDL-C and higher LDL-C levels (both *p* < 0.001), yielding substantially higher TG/HDL-C and LDL/HDL-C ratios (both *p* < 0.001). Furthermore, the HTG-AP group displayed worse glycemic indices (fasting glucose and HbA1c; both *p* < 0.001) and lower thyroid hormone levels (FT4 and FT3; both *p* < 0.001), although TSH levels did not differ between groups. Hepatobiliary markers (bilirubin and liver enzymes) were lower in patients with HTG-AP, consistent with the different etiological distributions (Table 2).

### Severity scores and clinical outcomes

Table 3 summarizes the between-group comparisons of severity scoring systems. Patients with HTG-AP had higher BISAP scores (1.52 ± 1.05 vs. 0.99 ± 0.81; *p* < 0.001) and MCTSI scores (3.24 ± 1.62 vs. 2.52 ± 1.26; *p* < 0.001). The qSOFA scores were also higher in this group (*p* = 0.002), whereas Ranson scores did not differ significantly between groups (*p* = 0.180) (Table 3). The composite adverse outcome occurred more frequently in patients with HTG-AP than in those with NHTG-AP (28.1% vs. 15.0%; *p* = 0.008). Among the components of this outcome, persistent organ failure (≥48 h) was more common in the HTG-AP group (18.8% vs. 8.6%; *p* = 0.015), as was prolonged ICU stay >7 days (14.8% vs. 6.4%; *p* = 0.023). The rate of necrosis requiring intervention did not differ significantly between groups (14.8% vs. 8.6%; *p* = 0.103). HTG-AP was also associated with a longer hospital length of stay (median 10 vs. 8 days; *p* = 0.007) and a higher incidence of pancreatic necrosis (18.8% vs. 10.0%; *p* = 0.036). In-hospital mortality remained low in both groups (3.1% vs. 1.4%; *p* = 0.426) (Table 1).

### Predictive model development and validation

Table 4 presents the multivariable modeling results. We retained BISAP and MCTSI in the final model using LASSO-regularized selection followed by multivariable logistic regression. Both BISAP (adjusted odds ratio [OR] = 8.42, 95% CI 2.89–24.51; *p* < 0.001) and MCTSI (adjusted OR = 1.68, 95% CI 1.21–2.33; *p* = 0.002) were independently associated with the composite adverse outcome (Table 4).

**Table 4:** Multivariable logistic regression analysis identifying independent predictors of the composite adverse outcome

Variable	β coefficient	Standard error	Wald χ^2^	p-value	OR	95% CI	VIF^†^
Final model (LASSO-selected multivariable logistic regression)							
BISAP score	2.131	0.546	15.24	<0.001	8.42	2.89–24.51	1.62
MCTSI score	0.519	0.168	9.55	0.002	1.68	1.21–2.33	1.62
Constant	-4.121	0.623	43.70	<0.001	0.016	0.005–0.053	—

Model performance: AUC (temporal validation) = 0.843 (95% CI: 0.762–0.924); optimism-corrected C-statistic = 0.831 (bootstrap 2000 iterations). BISAP: Bedside Index for Severity in Acute Pancreatitis; MCTSI: Modified CT Severity Index; OR: Odds Ratio; VIF: Variance Inflation Factor; CI: confidence interval.

**Figure 2:**
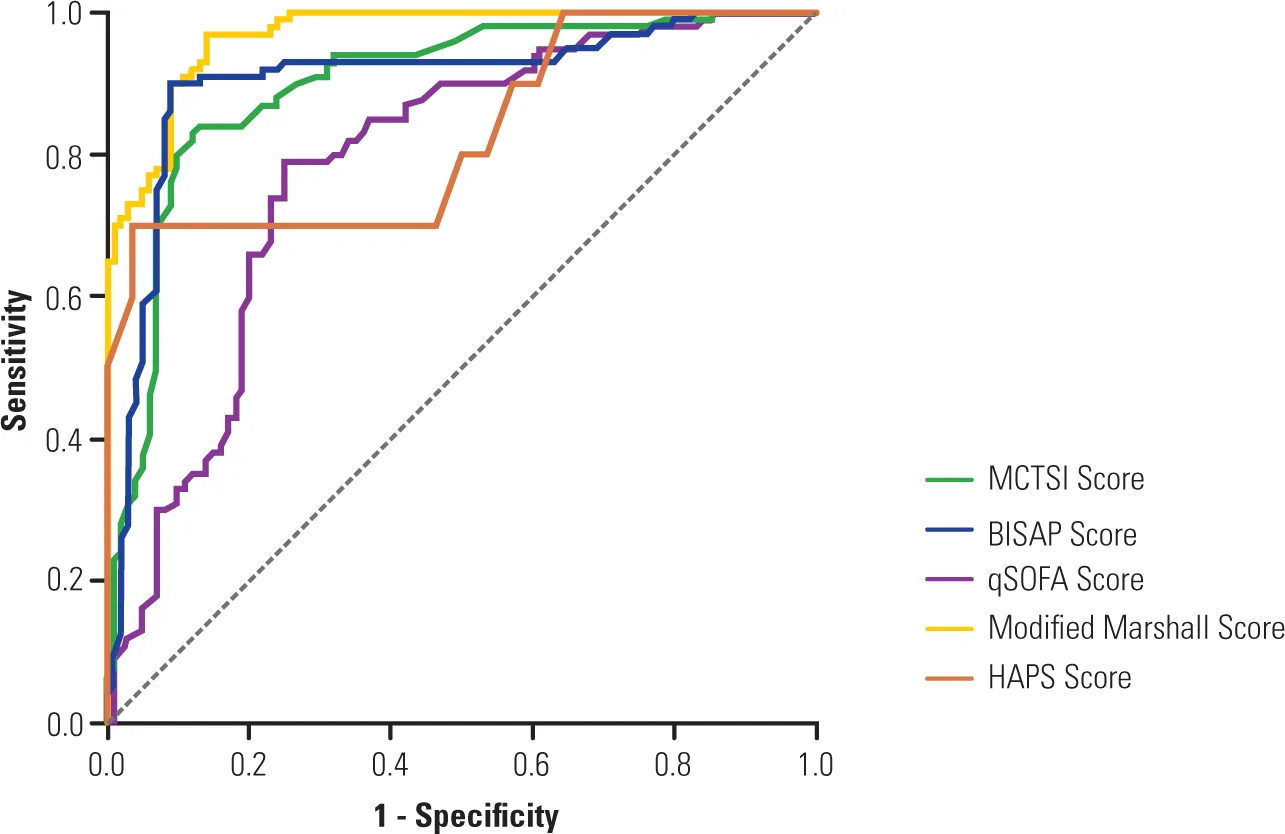
Receiver operating characteristic curves comparing the predictive performance of severity scoring systems for composite adverse outcomes.

Table 3 and Figure 2 summarize the discriminative performance of each score and the combined model. BISAP achieved the highest AUC among the individual scores, and the combined BISAP-MCTSI model demonstrated improved discrimination. Following Bonferroni correction for pairwise comparisons, the combined model outperformed the Ranson, qSOFA, and HAPS scores, although its difference compared with the Modified Marshall score was not statistically significant (Figure 2, Table 3).

**Figure 3:**
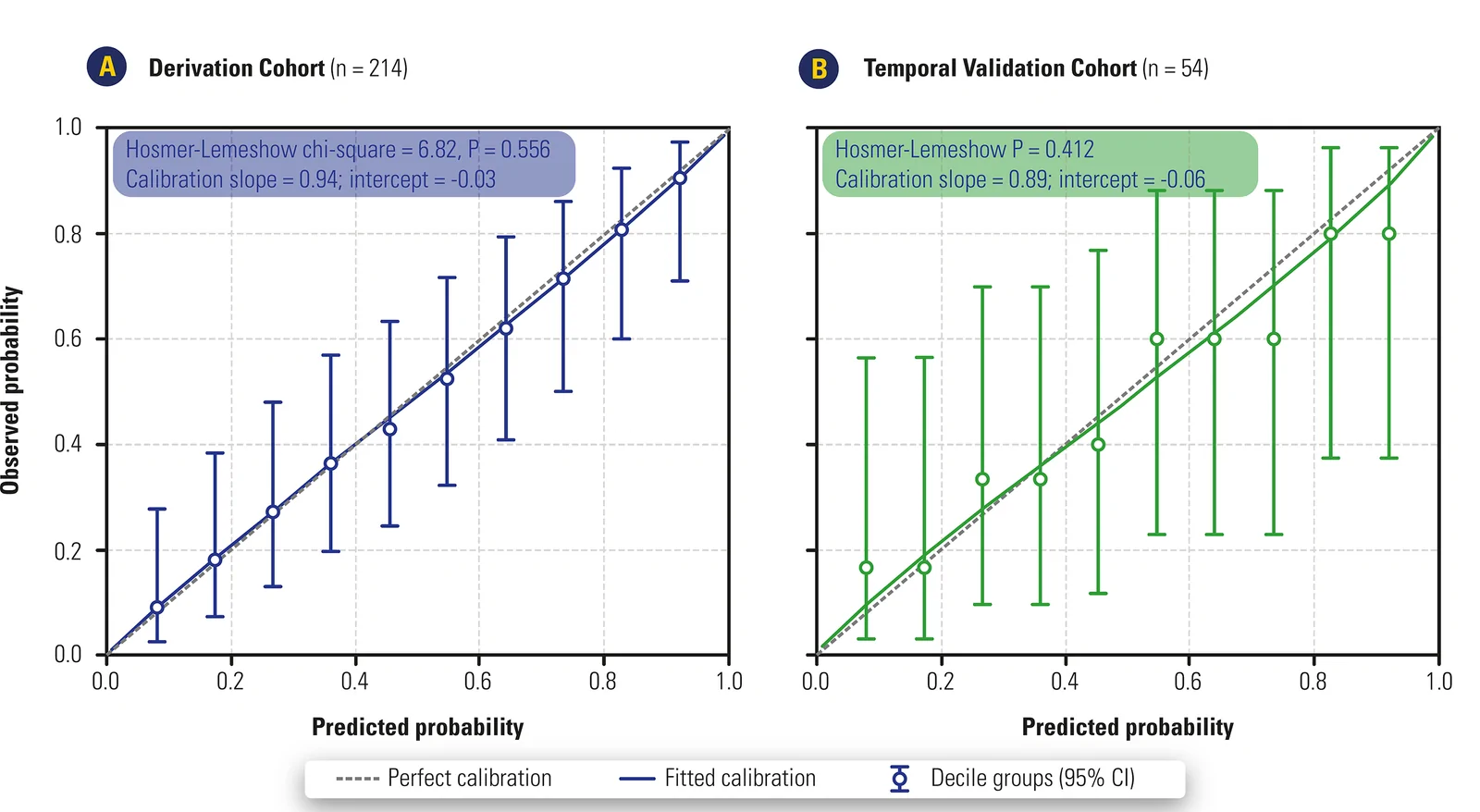
Calibration plots of the combined BISAP-MCTSI predictive model for the composite adverse outcome. (**A**) Derivation cohort (n = 214). Hosmer-Lemeshow test: χ^2^ = 6.82, *p* = 0.556; calibration slope = 0.94, intercept = −0.03. (**B**) Temporal validation cohort (n = 54). Hosmer-Lemeshow test: *p* = 0.412; calibration slope = 0.89, intercept = −0.06. The diagonal line represents perfect calibration. Data points represent decile groups of predicted risk with 95% confidence intervals.

We assessed the calibration of the BISAP-MCTSI model using calibration plots and the Hosmer-Lemeshow goodness-of-fit test. In the derivation cohort, the Hosmer-Lemeshow test was non-significant (χ^2^ = 6.82, df = 8, *p* = 0.556), indicating no evidence of poor fit. The calibration plot showed close agreement between the predicted and observed probabilities across the deciles of predicted risk (calibration slope = 0.94, intercept = −0.03). In the temporal validation cohort, calibration remained adequate (Hosmer-Lemeshow *p* = 0.412; calibration slope = 0.89, intercept = −0.06), albeit with an expected slight attenuation. Figure 3 provides the calibration plots for both cohorts.

To facilitate clinical application, we derived the optimal cutoff for the combined BISAP-MCTSI predicted probability using Youden’s index in the derivation cohort and subsequently evaluated it in the temporal validation cohort. The optimal probability threshold was 0.22 (corresponding approximately to a BISAP ≥ 2 combined with an MCTSI ≥ 4). In the temporal validation cohort, this threshold yielded a sensitivity of 82.1% (95% CI: 70.2–90.8%), a specificity of 78.4% (95% CI: 72.1–83.9%), a positive predictive value of 41.8% (95% CI: 32.6–51.5%), a negative predictive value of 95.8% (95% CI: 91.9–98.2%), a positive likelihood ratio of 3.80, and a negative likelihood ratio of 0.23.

For clinical practicability, we propose a simplified three-tier risk stratification scheme based on the two component scores:

1. Low risk (BISAP 0–1 AND MCTSI 0–2): Predicted adverse outcome probability <10%; ward-level monitoring with standard reassessment may be appropriate.
2. Intermediate risk (BISAP ≥ 2 OR MCTSI 3–4): Predicted probability 10–30%; close monitoring with clinical and biochemical reassessment at 24–48 hours is recommended.
3. High risk (BISAP ≥2 AND MCTSI ≥4): Predicted probability >30%; early ICU admission or step-up care should be considered.

These cutoffs are intended as clinical guidance pending external validation; they should be interpreted within the overall clinical context, including patient comorbidities, clinical trajectory, and institutional resources.

### Subgroup and sensitivity analyses

Table 5 provides the subgroup and sensitivity analyses of the BISAP-MCTSI model. Model discrimination remained high in both the HTG-AP and NHTG-AP subgroups (AUC, 0.902 vs. 0.864; interaction *p* = 0.186). Performance also remained consistent across the diabetes and obesity strata, as well as across the admission time subgroups. Sensitivity analyses, including a complete-case analysis, the inclusion of equivocal-etiology cases, and an expanded admission window, yielded similar AUCs, supporting the robustness of the model (Table 5).

**Table 5.** Subgroup and sensitivity analyses of the BISAP-MCTSI predictive model performance

Analysis type	Subgroup/condition	n	AUC (95% CI)	Sensitivity (%)	Specificity (%)	*p*-value for interaction/comparison
Primary subgroup analyses						
Etiology	HTG-AP	128	0.902 (0.842–0.962)	85.3	82.6	0.186
	NHTG-AP	140	0.864 (0.789–0.939)	80.9	79.8	
Diabetes status	Diabetic patients	92	0.893 (0.824–0.962)	83.7	81.2	0.542
	Non-diabetic patients	176	0.881 (0.815–0.947)	82.4	80.5	
Obesity status	Obese (BMI ≥30)	115	0.879 (0.808–0.950)	81.8	79.4	0.634
	Non-obese (BMI <30)	153	0.892 (0.829–0.955)	83.9	81.7	
Time to admission	0–12 hours	98	0.895 (0.828–0.962)	84.2	82.1	0.287
	12–24 hours	116	0.878 (0.810–0.946)	81.6	79.8	
Sex	Male	189	0.882 (0.821–0.943)	82.5	80.3	0.724
	Female	79	0.896 (0.826–0.966)	84.1	82.4	
Disease severity	Mild AP	168	0.854 (0.778–0.930)	79.8	78.2	0.312
	Moderately severe/severe AP	100	0.908 (0.852–0.964)	86.4	83.5	
Etiological overlap analyses						
Mixed etiology subgroup	Patients with ≥2 risk factors	47	0.856 (0.756–0.956)	79.2	77.1	—
Excluding mixed etiologies	Single etiology only	221	0.879 (0.812–0.946)	82.8	80.6	0.623^§^
Sensitivity analyses						
Missing data handling	Complete-case analysis (no imputation)	248	0.884 (0.825–0.943)	82.6	80.4	0.756^§^
	Original analysis (with imputation)	268	0.887 (0.828–0.946)	83.1	80.9	
Etiology classification	Including equivocal-etiology patients	280	0.881 (0.823–0.939)	82.2	79.8	0.623^§^
	Excluding equivocal-etiology patients	268	0.887 (0.828–0.946)	83.1	80.9	
Admission time window	0–72 hours admission	312	0.869 (0.812–0.926)	80.5	78.6	0.184^§^
	0–24 hours admission (primary)	268	0.887 (0.828–0.946)	83.1	80.9	
Outcome definition	Necrosis >30% threshold	268	0.892 (0.835–0.949)	83.8	81.4	0.714^§^
Propensity score matching	Matched HTG-AP vs NHTG-AP	78 pairs (n=156)	0.875 (0.802–0.948)	82.0	79.5	0.012^††^

HTG-AP: hypertriglyceridemic acute pancreatitis; NHTG-AP: non-hypertriglyceridemic acute pancreatitis; AUC: area under the receiver operating characteristic curve; CI: confidence interval; BMI: body mass index; AP: acute pancreatitis; OR: odds ratio; BISAP: Bedside Index for Severity in Acute Pancreatitis; MCTSI: Modified CT Severity Index.

Sensitivity and specificity are reported at the optimal Youden’s index cutoff for each subgroup.

P-values for interaction were derived from logistic regression models including a subgroup × BISAP–MCTSI predicted probability interaction term.

§ P-value for comparison of AUC between sensitivity analysis and primary analysis using DeLong’s test.

†† P-value for composite outcome rate comparison between propensity score–matched HTG-AP and NHTG-AP groups using McNemar’s test.

Propensity score matching was performed using 1:1 nearest-neighbor matching without replacement (caliper = 0.2 SD of the logit of the propensity score), adjusting for age, sex, BMI, diabetes mellitus, hypertension, and time from onset to admission. Standardized mean differences for all matched covariates were <0.10 after matching.

To evaluate the potential impact of etiological misclassification, we performed a sensitivity analysis excluding all 47 patients with overlapping etiologies (i.e., more than one identifiable risk factor). In this restricted cohort (n = 221; HTG-AP, n = 100; NHTG-AP, n = 121), the composite adverse outcome rate remained significantly higher in the HTG-AP group than in the NHTG-AP group (28.9% vs. 13.2%; *p* = 0.004). The BISAP-MCTSI model maintained robust discrimination (AUC = 0.879; 95% CI: 0.812–0.946), consistent with the primary analysis (AUC = 0.887). Furthermore, among the 47 patients with mixed etiologies, the composite adverse outcome rate was 25.5% (12/47), and the BISAP-MCTSI model demonstrated adequate discrimination within this subgroup (AUC = 0.856; 95% CI: 0.756–0.956). These findings indicated that etiological misclassification in overlap cases did not substantially drive the primary results (Table 5).

## DISCUSSION

This retrospective cohort study of 268 patients with AP yielded three principal findings. First, HTG-AP constituted a distinct clinical phenotype characterized by younger age, male predominance, higher BMI, greater recurrence rates, and worse in-hospital outcomes. Second, HTG-AP exhibited a characteristic early biochemical signature featuring heightened systemic inflammation, profound atherogenic dyslipidemia, disturbed glucose homeostasis, and lower circulating thyroid hormones. Third, the combined BISAP-MCTSI model provided superior prediction of the composite adverse outcome compared with several commonly used individual scoring tools. These findings supported etiology-aware, integrated risk stratification in AP.

### Comparison with existing literature

Our findings aligned with previous studies that have identified HTG-AP as an increasingly common subtype, particularly among younger patients (^18^). Previous studies have reported a rising incidence of HTG-AP in populations with a high prevalence of obesity, type 2 diabetes, and metabolic syndrome. These comorbid conditions contribute to elevated serum TG levels and remain well-established risk factors for HTG-AP (^19^,^20^).

Compared with biliary-induced pancreatitis, which commonly affects older adults, previous research has frequently associated HTG-AP with more severe systemic inflammation and an increased risk of early organ dysfunction (^21^). This aligned with our observation of elevated inflammatory biomarkers and metabolic ratios (TG/HDL, LDL/HDL) in the HTG-AP group, which indicated a more aggressive clinical course even in the early phase of the disease.

Previous studies have validated the BISAP score as a practical tool for the early identification of high-risk patients with AP because of its simplicity and reliance on readily available clinical parameters (^22^,^23^). Our findings reinforced this conclusion, demonstrating that the BISAP score, alongside the Modified Marshall score, remained a robust and independent predictor of severe disease within the specific context of HTG-AP. These results supported the broader applicability of these tools across etiological subtypes and highlighted their clinical utility in diverse patient populations.

Although both BISAP and MCTSI are well-established severity assessment tools for AP, and combining bedside clinical scores with imaging-based assessments is not unprecedented, the specific contribution of the present study merits clarification. To our knowledge, previous literature has not formally evaluated the combination of BISAP and MCTSI as an integrated predictive model for a composite adverse outcome in a cohort enriched for HTG-AP. Furthermore, no prior research has systematically compared its performance against four other commonly used scoring systems within this specific etiological context using LASSO-based variable selection, temporal validation, and rigorous multiple comparison correction. The principal contribution of this study lies not in proposing a novel scoring instrument per se, but rather in providing empirical evidence that this specific combination offers complementary prognostic information. Specifically, BISAP captured early systemic physiological derangement, and MCTSI captured local pancreatic and peripancreatic disease burden. Furthermore, our findings demonstrated that this combination maintained robust discriminative performance across both the HTG-AP and NHTG-AP subgroups (AUC, 0.902 vs. 0.864; interaction *p* = 0.186), an observation not previously reported.

### Clinical implications

These findings carry important clinical implications for the management of AP, particularly in patients with hypertriglyceridemia-induced disease. Given the younger age and higher recurrence rate observed in HTG-AP, early identification and close monitoring are essential, especially in patients presenting with recurrent episodes or known metabolic disorders. Our results support a two-tier approach to early risk stratification. For immediate bedside assessment, the BISAP score demonstrated the strongest individual predictive performance and can be calculated within 24 hours of admission using readily available clinical and laboratory data. When contrast-enhanced CT is performed—as is routine in many centers within the first 72 hours—incorporating the MCTSI into a combined BISAP-MCTSI model further improves prognostic accuracy. This combined approach outperformed Ranson, qSOFA, and HAPS after correction for multiple comparisons, and performed comparably to the Modified Marshall score, which has the inherent limitation of partial circularity when used to predict organ failure-defined outcomes.

Furthermore, lipid-related markers (e.g., TG/HDL-C and LDL/HDL-C ratios) may serve as additional risk stratification indicators in hypertriglyceridemic patients. Although not currently integrated into standard scoring systems, these ratios reflect the extent of metabolic disturbance and inflammatory burden and warrant further investigation as potential adjuncts in clinical decision-making. Rapid and accurate severity assessment is critical to guide early fluid resuscitation, ICU admission, and escalation of care when appropriate (^22^).

Building on the quantitative risk stratification results, the proposed three-tier classification (low/intermediate/high risk based on BISAP and MCTSI thresholds) provides a pragmatic framework for clinical decision-making. Notably, the high negative predictive value (95.8%) of the low-risk category may be particularly useful for identifying patients who can be safely managed in general ward settings, thereby optimizing resource allocation. Conversely, the high-risk category (BISAP ≥2 AND MCTSI ≥4), although associated with a moderate positive predictive value (41.8%), identifies a subgroup with more than a 30% probability of adverse outcomes, warranting proactive escalation of care. We emphasize that these thresholds require prospective external validation before formal incorporation into clinical protocols.

### Pathophysiological mechanisms underlying enhanced inflammation in HTG-AP

The heightened inflammatory response observed in HTG-AP is likely multifactorial. Markedly elevated TG increase circulating chylomicrons, and intrapancreatic hydrolysis of TG by pancreatic lipase generates excess free fatty acids (^24^). When albumin-binding capacity is exceeded, unbound free fatty acids exert direct cytotoxic effects on acinar cells and vascular endothelium, aggravating local pancreatic injury and increasing capillary permeability. Free fatty acids can also promote mitochondrial dysfunction and oxidative stress, amplifying inflammatory signaling (e.g., NF-κB activation) and downstream cytokine release (e.g., TNF-α, IL-1β, and IL-6) (^24^,^25^). In addition, hypertriglyceridemia increases blood viscosity and impairs pancreatic microcirculation, predisposing to microvascular ischemia and tissue hypoxia, which further augments systemic inflammation and may contribute to early organ dysfunction (^26^).

The lower FT4 and FT3 levels observed in HTG-AP are consistent with non-thyroidal illness syndrome, a common response to acute critical illness. This hormonal pattern may reflect inflammatory severity and altered peripheral thyroid hormone metabolism rather than primary thyroid disease; however, whether thyroid hormone changes are merely markers of severity or contribute mechanistically to disease progression requires prospective study.

### Limitations and future directions

Several limitations of this study merit consideration. First, the single-center, retrospective design conducted at a tertiary referral hospital introduces potential biases at multiple levels. Referral bias may have resulted in an overrepresentation of HTG-AP cases and disproportionately severe disease compared with the general AP population. Although standardized data abstraction forms with dual verification were employed, the information bias inherent to retrospective data extraction cannot be fully excluded. Furthermore, residual confounding by unmeasured variables, such as dietary habits, medication adherence, genetic predisposition to hypertriglyceridemia, and socioeconomic factors, may have influenced our results.

While multiple analytical strategies were used to mitigate confounding, including LASSO regularization, propensity score matching, and comprehensive subgroup and sensitivity analyses, these approaches cannot fully substitute for prospective randomization. Accordingly, external validation in multicenter, geographically diverse cohorts encompassing both tertiary and community hospital settings is essential before the BISAP-MCTSI model can be recommended for widespread clinical adoption. Although temporal split validation and bootstrap resampling provide some protection against overfitting, these internal validation approaches are not a substitute for true external validation in geographically and demographically distinct populations. To address this, we initiated a multicenter collaboration with three additional institutions spanning different geographic regions and healthcare settings. This effort aims to prospectively validate the BISAP-MCTSI model in an independent cohort of approximately 500 patients with AP, with results anticipated within 18 months. Until external validation is completed, the current findings should be considered hypothesis-generating, and the proposed risk stratification scheme should be applied with clinical judgment rather than treated as a definitive decision rule.

Second, the temporal validation cohort comprised only 54 patients with an estimated 11 to 12 composite outcome events, which limits the precision of the validation AUC estimate (95% CI: 0.762–0.924) and the statistical power of the calibration assessment. Consequently, the non-significant Hosmer-Lemeshow test (*p* = 0.412) in this small sample should be interpreted cautiously, as this test has limited power to detect miscalibration when event counts are low. Larger prospective validation studies with adequate event rates are needed to confirm the model’s calibration and discrimination.

Third, the MCTSI relies on contrast-enhanced CT and is therefore inherently influenced by the timing of image acquisition and interobserver variability in radiological interpretation. In clinical practice, contrast-enhanced CT is not uniformly performed within a standardized time window, which may introduce heterogeneity into MCTSI scoring. Future multicenter studies incorporating standardized imaging protocols, predefined scan timing, and reproducibility assessments (e.g., automated or semi-automated imaging metrics) would strengthen the generalizability and reliability of this scoring component.

Fourth, we focused exclusively on in-hospital outcomes and did not assess longer-term endpoints, such as recurrent pancreatitis, the development of endocrine or exocrine pancreatic insufficiency, post-discharge mortality, or quality of life. Given the notably high recurrence rate observed in the HTG-AP group (39.8%), future prospective cohorts should incorporate longitudinal follow-up to evaluate whether early triglyceride-lowering strategies (e.g., fibrate therapy, insulin infusion, or plasmapheresis) and comprehensive metabolic risk modification can reduce disease recurrence and improve long-term outcomes.

Fifth, although a standardized hierarchical adjudication protocol with independent review was employed, and sensitivity analyses excluding patients with overlapping etiologies yielded consistent results, etiological classification in AP remains inherently imperfect when multiple risk factors coexist. In the present study, 47 patients (17.5%) had more than one identifiable etiological factor. While inter-rater agreement was excellent (κ = 0.89), misclassification cannot be entirely excluded. Future multicenter studies employing prospective, protocolized etiological assessments, preferably incorporating genetic testing for familial hypertriglyceridemia, standardized quantification of alcohol consumption using validated instruments, and systematic biliary evaluation, would further enhance classification accuracy and strengthen causal inference.

Sixth, although we adopted a composite endpoint specifically designed to mitigate circularity concerns, the potential overlap between predictor and outcome components warrants acknowledgment. The BISAP score incorporates SIRS criteria, which partially overlap with the clinical parameters used to define organ failure. Notably, the LASSO variable selection procedure did not retain the modified Marshall score, whose components directly define one element of the composite outcome (persistent organ failure), thereby providing some reassurance against circular prediction. Nevertheless, this inherent limitation should be considered when interpreting the predictive performance of the BISAP score. Future studies may benefit from employing strictly outcome-independent predictors or alternative endpoint definitions to further address this concern.

Lastly, missing data affected 20 patients (7.5%) in the primary analysis. Although imputation was performed and a complete-case sensitivity analysis (n = 248) yielded concordant results (AUC = 0.884 vs. 0.887), the impact of missing data on model performance cannot be entirely excluded. Future prospective studies utilizing protocolized data collection will help minimize this concern.

In conclusion, HTG-AP is a metabolically distinct subtype of acute pancreatitis characterized by younger age, higher BMI, greater disease recurrence, and a stronger inflammatory response; furthermore, it is associated with a higher risk of adverse in-hospital outcomes. For early risk stratification, a combined BISAP-MCTSI approach demonstrated better predictive performance than several commonly used tools, supporting integrated bedside and imaging-based assessments when CT is available. Additionally, lipid-related indices may provide further prognostic information and warrant continued validation.

## Data Availability

the authors are willing to share all data related to the study upon reasonable request.

## Supplementary Material

**Supplementary Table S1.** Definitions, components, score ranges, and assessment time windows of severity scoring systems

Scoring System	Components	Score Range	Assessment Time Window	High-Risk Threshold	Reference
BISAP	BUN >25 mg/dL; impaired mental status (GCS <15); SIRS (≥2 criteria); age >60 years; pleural effusion	0-5	Within 24 h of admission	≥3	^Wu et al., 2008^
MCTSI	Pancreatic inflammation (0/2/4); pancreatic necrosis (0/2/4); extrapancreatic complications (0/2)	0-10	At time of CECT (typically within 72 h)	≥4	^Mortele et al., 2004^
Modified Marshall	Respiratory (PaO2/FiO2); renal (creatinine); cardiovascular (SBP) - each scored 0-4	0-4 per system	Within 24 h; reassessed at 48 h for persistence	≥2 in any system	^Banks et al., 2013^
Ranson’s Criteria	5 criteria at admission + 6 criteria at 48 h (see Methods for details)	0-11	Admission + 48 h	≥3	^Ranson et al., 1974^
qSOFA	Altered mental status (GCS <15); SBP <=100 mmHg; RR ≥22/min	0-3	Within 24 h of admission	≥2	Singer et al., 2016
HAPS	No rebound tenderness/guarding; normal hematocrit; normal creatinine	0-3	Within 24 h of admission	=3 (harmless)	^Lankisch et al., 2009^

BISAP: Bedside Index for Severity in Acute Pancreatitis; BUN: blood urea nitrogen; GCS: Glasgow Coma Scale; SIRS: systemic inflammatory response syndrome; MCTSI: Modified CT Severity Index; CECT: contrast-enhanced computed tomography; SBP: systolic blood pressure; PaO2: partial pressure of arterial oxygen; FiO2: fraction of inspired oxygen; qSOFA: quick Sequential Organ Failure Assessment; RR: respiratory rate; HAPS: Harmless Acute Pancreatitis Score.

**Supplementary Table S2.** Spearman correlation matrix of six severity scoring systems (n = 268)

	BISAP	MCTSI	Modified Marshall	Ranson	qSOFA	HAPS
BISAP	1.00	0.52	0.68	0.46	0.58	-0.44
MCTSI	0.52	1.00	0.49	0.42	0.38	-0.36
Modified Marshall	0.68	0.49	1.00	0.44	0.62	-0.48
Ranson	0.46	0.42	0.44	1.00	0.35	-0.32
qSOFA	0.58	0.38	0.62	0.35	1.00	-0.41
HAPS	-0.44	-0.36	-0.48	-0.32	-0.41	1.00

Values represent Spearman rank correlation coefficients (rho).

All correlations were statistically significant at P < 0.01 (two-sided) except where noted.

HAPS scores are inversely correlated with the other scoring systems because higher HAPS scores indicate a predicted “harmless” (non-severe) course, whereas higher scores on the other systems indicate greater severity.

BISAP: Bedside Index for Severity in Acute Pancreatitis; MCTSI: Modified CT Severity Index; qSOFA: quick Sequential Organ Failure Assessment; HAPS: Harmless Acute Pancreatitis Score.

**Supplementary Table S3.** Distribution of coexisting etiological risk factors by study group

Coexisting Etiological Factor	Total (n=268)	HTG-AP (n=128)	NHTG-AP (n=140)	Chi-square/Fisher’s	P-value
Any coexisting etiological factor, n (%)	47 (17.5)	28 (21.9)	19 (13.6)	chi-square = 3.195	0.074
TG ≥5.65 mmol/L + alcohol use, n (%)	30 (11.2)	18 (14.1)	12 (8.6)	chi-square = 2.053	0.152
TG ≥5.65 mmol/L + biliary disease, n (%)	14 (5.2)	8 (6.3)	6 (4.3)	chi-square = 0.529	0.467
TG ≥5.65 mmol/L + alcohol + biliary, n (%)	3 (1.1)	2 (1.6)	1 (0.7)	Fisher’s exact	0.607
Single identifiable etiology only, n (%)	221 (82.5)	100 (78.1)	121 (86.4)	chi-square = 3.195	0.074

Coexisting etiological factors refer to the presence of more than one identifiable risk factor for acute pancreatitis in the same patient (e.g., hypertriglyceridemia plus significant alcohol consumption, or hypertriglyceridemia plus biliary disease).

The predominant etiology for each patient was assigned using the hierarchical adjudication protocol described in the Methods section.

TG: triglycerides; HTG-AP: hypertriglyceridemic acute pancreatitis; NHTG-AP: non-hypertriglyceridemic acute pancreatitis.

*P*-values were calculated using chi-square test or Fisher’s exact test as appropriate.
